# Generic stress rectification in nonlinear elastic media†

**DOI:** 10.1039/d2sm01606k

**Published:** 2023-04-04

**Authors:** Félix Benoist, Guglielmo Saggiorato, Martin Lenz

**Affiliations:** a Université Paris-Saclay, CNRS, LPTMS 91400 Orsay France martin.lenz@universite-paris-saclay.fr fbenoist@igc.gulbenkian.pt; b PMMH, CNRS, ESPCI Paris, PSL University, Sorbonne Université, Université de Paris, F- 75005 Paris France

## Abstract

Stress propagation in nonlinear media is crucial in cell biology, where molecular motors exert anisotropic force dipoles on the fibrous cytoskeleton. While the force dipoles can be either contractile or expansile, a medium made of fibers which buckle under compression rectifies these stresses towards a biologically crucial contraction. A general understanding of this rectification phenomenon as a function of the medium's elasticity is however lacking. Here we use theoretical continuum elasticity to show that rectification is actually a very general effect in nonlinear materials subjected to anisotropic internal stresses. We analytically show that both bucklable and constitutively linear materials subjected to geometrical nonlinearities rectify small forces towards contraction, while granular-like materials rectify towards expansion. Using simulations, we moreover show that these results extend to larger forces. Beyond fiber networks, these results could shed light on the propagation of stresses in brittle or granular materials following a local plastic rearrangement.

## Abstract 2

In the cytoskeleton, the buckling of fibers rectifies internal stresses towards contraction. We use theoretical continuum elasticity to show that rectification is a general effect in nonlinear materials subjected to anisotropic internal stresses.

## Graphical abstract

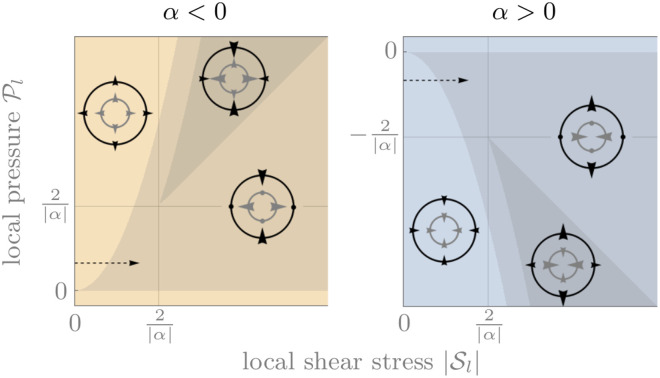

## Introduction

The active, stress-generating role of many biological materials stems from their ability to transmit internally generated forces. In cells, the action of molecular motors and the growth of protein fibers over a few nanometers generates anisotropic forces that are further transmitted by a fibrous network, the cytoskeleton, to the scale of the whole cell.^1,2^ At larger length scales, polarized cells in connective tissues exert anisotropic stresses on another fibrous network, the extracellular matrix, which again propagates these stresses far from their application point.^3,4^

The well-characterized nonlinear stress response of these networks^5–7^ plays a crucial role in force transmission, allowing for the enhancement of contractile stresses^8–11^ and promoting long-range mechano-sensitivity.^12–16^ Beyond this quantitative stress amplification, the nonlinear response of fiber networks also leads to qualitative changes in the propagated stresses,^19^ as previously shown in numerical simulations.^9^ In these simulations, a localized active unit exerts anisotropic forces in the center of a large network of discrete fibers, each of which can buckle under a sufficiently large compressive force. For localized forces much larger than this buckling threshold, the far-field stresses transmitted by the network become contractile. This is valid even in cases where the local forces are predominantly expansile, because the network resists and therefore propagates tension more than compression. This stress “rectification” has strong implications for biological force propagation, and could be one of the reasons why the actomyosin cytoskeleton is overwhelmingly observed to contract irrespective of its detailed internal architecture.

Here, we generalize these results beyond bucklable fiber networks, and demonstrate that stress rectification is a generic corollary of stress propagation in a nonlinear elastic medium. Our approach is based on a continuum formalism that allows a general discussion of arbitrary nonlinearities. We consider both geometrical nonlinearities and generic material-dependent nonlinearities describing the response of the material to compression or tension. Nonlinearities whereby the material stiffens under tension and soften under compression are characteristic of bucklable fiber networks.^6^ For the sake of suggestiveness, here we loosely refer to such materials as “bucklable” irrespective of the microscopic origin of this nonlinearity. This origin may or may not involve thermal fluctuations depending on the system considered. In contrast, materials that soften under tension and stiffen under compression, or “anti-buckle”, may offer a description of granular media, where contacts between grains are disrupted as the confining pressure is decreased.^17^ Under shear, these materials experience localized plastic events known as shear transformations which generate anisotropic internal stresses similar to those induced by molecular motors in the cytoskeleton.^18^ We show that the elastic constants describing the weakly nonlinear response of these materials are a reliable predictor of the sign and magnitude of rectification.

We consider a piece of homogeneous, isotropic elastic medium of dimension *d* comprised in a domain Ω. A set of anisotropic “active units” (*e.g.*, molecular motors or shear transformation zones) exerts forces and/or imposes local displacements on the medium. This induces a force density **f**, resulting in a Cauchy stress tensor ***σ*** given by the force balance equation *f*_*i*_ = −∂*σ*_*ij*_/∂*X*_*j*_ (summary of notation in Table 1). Here **X** = **x** + **u** is the final location (in the “target space”) of a material point initially located in **x** (in the “initial space”), **u** denotes the displacement vector and the summation over repeated indices is implied. The boundary ∂*Ω* of the medium is held fixed, such that the forces exerted by the active units are transmitted through the medium and cause it to exert a coarse-grained stress1
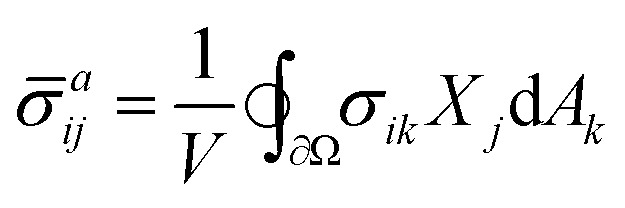
onto the boundary,^19^ where *V* is the volume of the medium and d**A** the outward-directed area element in the target space. In the context of active matter, ***σ̄***^*a*^ is known as the active stress generated by the overall system comprised by the medium and the active units.^20^ We define as contraction (expansion) a situation where the active pressure *P*_*a*_ = −*σ̄*^*a*^_*ij*_/*d* is negative (positive). To investigate the relationship between the local forces **f** and the active stress ***σ̄***^*a*^, we define the coarse-grained local stress2
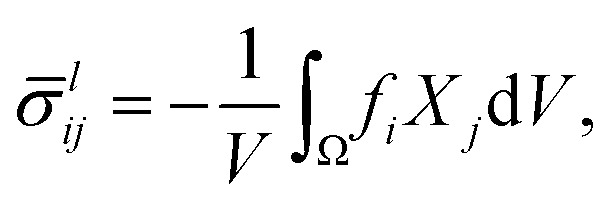
where d*V* is the volume element in the target space. In the special case where the force transmission is entirely linear, this relation simply reads ***σ̄***^*a*^ = ***σ̄***^*l*^, implying in particular an equality of the active and local pressures *P*_*a*_ = *P*_*l*_ = −*σ̄*^*l*^_*ii*_/*d*. In that case, contractile (expansile) local forces always imply a contractile (expansile) active stress. These equalities are however violated in nonlinear media,^19,21^ and the local and active pressures *P*_*l*_ and *P*_*a*_ can have opposite signs. We show here that this stress rectification may arise from geometrical and/or constitutive nonlinearities in the material's elastic response, and that geometrical nonlinearities always bias the system towards contraction. We then investigate the effect of generic, lowest-order constitutive nonlinearities, and characterize the regimes conducive to rectification towards contraction and expansion. Finally, we use finite-element simulations to show that our conclusions remain qualitatively valid at higher orders.

**Table 1:** Summary of notation

Name	Meaning
x	Position in the initial space
**X**	Position in the target space
*r*_in_, *r*_out_	Inner and outer radii
*V*, **A**	Volume and oriented area in the target space
*d*	Dimension
*e*_0_, *e*_2_	Components of the imposed displacement
**u**	Displacement vector
***η***	Displacement gradient
***ε***	Green–Lagrange strain tensor
*I*, *J*	Invariants of the strain tensor
*E*	Elastic energy density
*K*, *G*	Differential bulk and shear moduli
*κ*, *μ*	Linear bulk and shear moduli
*ν*	Poisson ratio
*κ*_1_, *μ*_1_	Corrections to the elastic moduli
**f**	Force density
***σ***	Cauchy stress tensor
***σ̄*** ^*a*^ (***σ̄***^*l*^)	Coarse-grained active (local) stress tensor
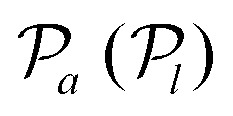	Coarse-grained active (local) pressure
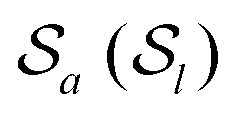	Coarse-grained active (local) shear stress

## Results

We describe the elastic deformation of our medium using the displacement gradient *η*_*ij*_ = ∂*u*_*i*_/∂*x*_*j*_ and introduce the Green–Lagrange strain tensor ***ε*** = (***η*** + ***η***^T^ + ***η***^T^*η*)/2.^22^ The last, nonlinear term of ***ε*** is purely geometrical and accounts for, *e.g.*, material rotations. We express the Cauchy stress as a function of the elastic energy density *E* in the initial space by 
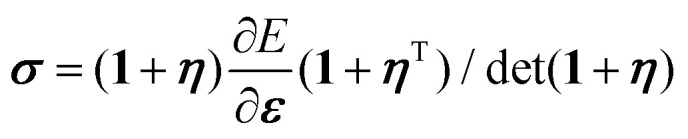
, where **1** denotes the unit tensor. Our choice of deriving the elastic stress from an energy density implicitly rules out odd elastic materials, where a more general stress dependence emerges from *e.g.*, the presence of active, energy-consuming elements within the elastic material.^23^ By contrast, here we explicitly model any active elements through the force density **f**, which we embed in an otherwise passive material.

### Rectification due to geometric nonlinearities

We first consider a constitutively linear material with a quadratic energy density *E* = *κε*_*ii*_^2^/2 + *μ*(*ε*_*ij*_^2^ − *ε*_*ii*_^2^/*d*), where *κ* and *μ* are the bulk and shear moduli. We use the divergence theorem to turn the right-hand side of eqn (1) into a volume integral, and combine the expression of the Cauchy stress, the force balance equation and eqn (2) to find3
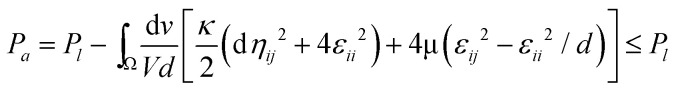
where the integral runs over the initial space. The inequality in eqn (3) is proven in the ESI† and means that the system as a whole is always more contractile than the local forces, implying that geometrical nonlinearities always induce a rectification towards contraction.

### Parametrization of weak constitutive nonlinearities

To describe nonlinearities resulting from the medium's constitutive properties, we consider a two-dimensional isotropic, achiral elastic medium with a non-harmonic energy density:4
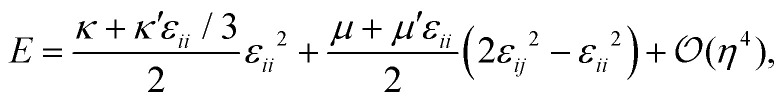
where the coefficients *κ*′, *μ*′ can be of either sign and characterize the most general, lowest-order nonlinearity. According to eqn (4), when the material is isotropically dilated by a relative amount *ε*_*ii*_ ∼ δ*V*/*V*_0_ its bulk (shear) modulus exceeds that of a purely harmonic material by *κ*′*δV*/*V*_0_ (*μ*′*δV*/*V*_0_). More generally, we may consider a combination of bulk expansion and simple shear5
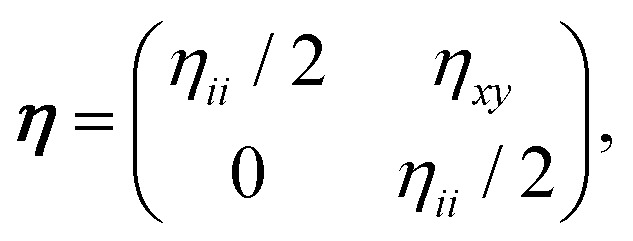
compute the Cauchy stress tensor, and derive the differential bulk and shear moduli as6a
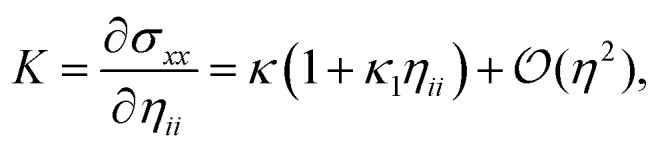
6b
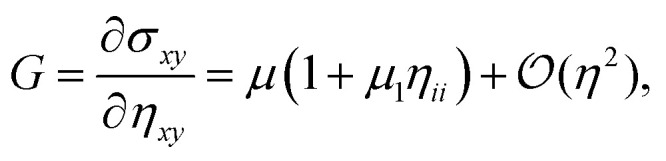
where the first order nonlinear corrections to the moduli7a
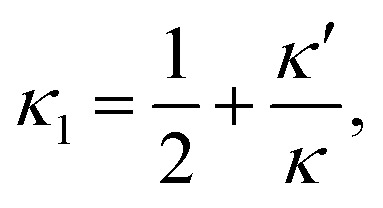
7b
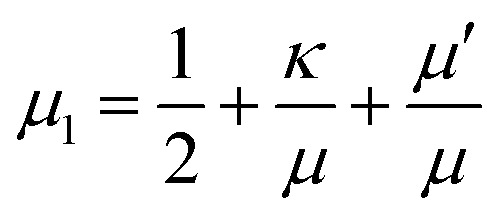
include contributions from geometrical as well as constitutive nonlinearities.

Based on rheology measurements, we estimate *κ*_1_ ≈ 100 and *μ*_1_ ≈ 30 for gels of the extracellular matrix filaments fibrin and collagen.^24^ These positive values are consistent with the notion that biological fiber networks buckle, and therefore soften, under compression (*η*_*ii*_ < 0). In contrast, granular materials tend to increase their cohesion under compression. Experiments and simulations on polydisperse soft spheres near jamming thus suggest *κ*_1_ ≈ 0 and *μ*_1_ ∈ [−400, −4] (see ref. 25, 26 and ESI†). An intermediate behavior is observed in fiber networks with stiff grain-like inclusions mimicking connective tissues. This gives rise to a more complicated sign combination which depends on the inclusion density.^27,28^ Qualitatively, we expect systems with an increasing density of inclusion to smoothly interpolate between the rectification behaviors of fibrous and granular materials. Finally, a standard “neo-Hookean” model of rubber displays *κ*_1_ > 0 and *μ*_1_ < 0 with small values,^29,30^ see below.

### Rectification in a simple circular geometry

**Fig. 1:**
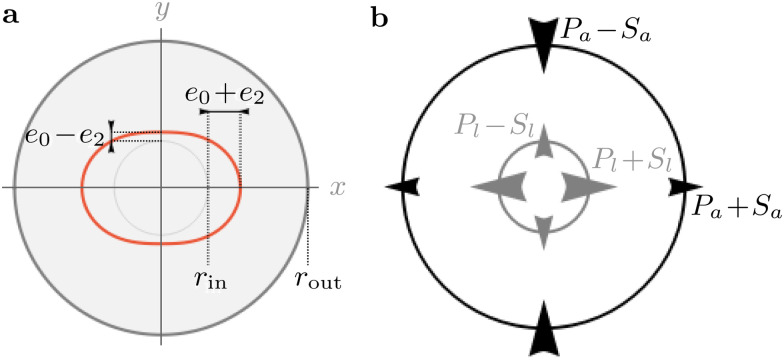
Sketches of the imposed anisotropic displacement and the resulting coarse-grained stresses. (a) In the target configuration, the inner light-gray circle with radius *r*_in_ is moved to the orange ring. (b) Stress components in a particular situation where the local pressure *P*_*l*_ is positive and the active pressure *P*_*a*_ at the boundary is negative.

To explicitly predict the active pressure resulting from rectification, we consider a simple circular piece of elastic medium with radius *r*_out_ and a single active unit at its center. The active unit is a circle with radius *r*_in_ at rest, and undergoes a radial displacement [Fig. 1a]

8 **u**(*r*_in_) = *r*_in_[*e*_0_ + *e*_2_ cos(2*θ*)] **r̂**

This induces a mixture of compression, tension and shear on the medium. Symmetry imposes that the local and active stress tensors take the form9
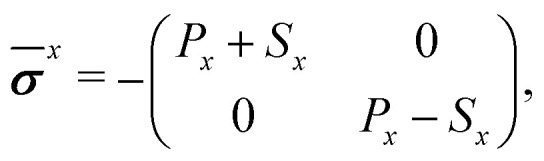
in Cartesian coordinates, for *x* ∈ {*l*,*a*} [Fig. 1b]. As shown in eqn (2), the local coarse-grained stress ***σ̄***^*l*^ is the ratio of a force dipole by the volume *V*. Assuming a constant local dipole, ***σ̄***^*l*^ thus decreases with increasing system size *V* due to dilution. A similar statement holds for ***σ̄***^*a*^. It is thus useful for our discussion to define the quantities 
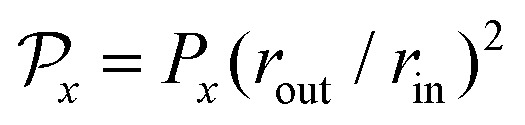
 and 
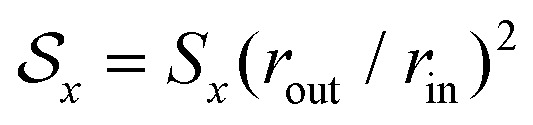
 which are not subject to this dilution. In this sense, they behave as force dipole components. In the following, we consider the lowest order in the weakly nonlinear regime *e*_0_, *e*_2_ ≪ 1 (see ESI†).

We perturbatively solve the force balance equation using eqn (8) as well as the fixed boundary condition in *r*_out_ to compute the pressure and shear components 
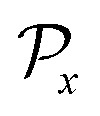
, 
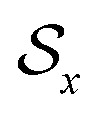
 as10a
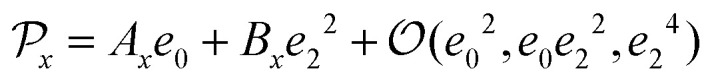
10b
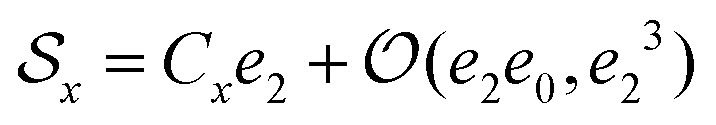
where the full expressions of *A*_*x*_, *B*_*x*_ and *C*_*x*_ as functions of the properties of the medium is very cumbersome and presented only in the ESI.† As an illustration, in the case *μ* = *κ*/2 (characteristic, *e.g.*, of a triangular spring lattice) and to leading order in the limit of a large piece of elastic medium, these expressions yield11a
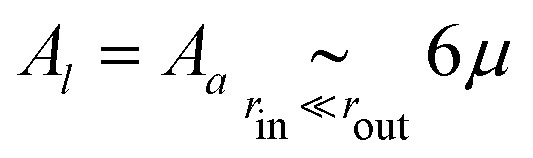
11b
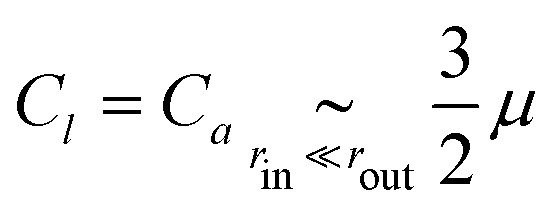
11c
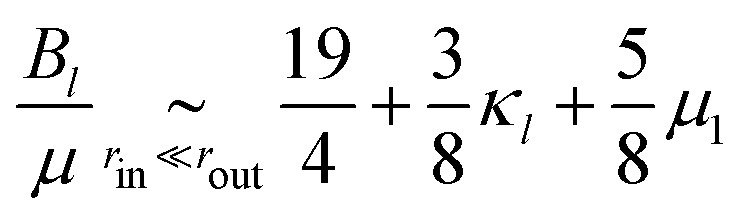
11d
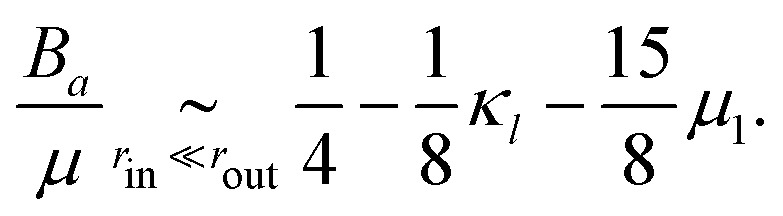


The active stresses can then be computed from the local ones through12
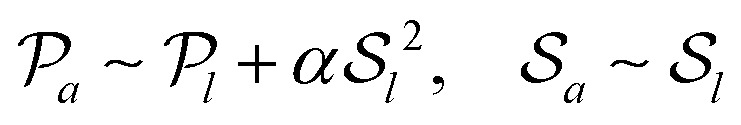
where *αμ* = *μ*(*B*_*a*_ − *B*_*l*_)/*C*_*l*_^2^ is a dimensionless function of *r*_out_/*r*_in_, *κ*/*μ*, *κ*_1_ and *μ*_1_. In the special case considered in eqn (11), it reads13
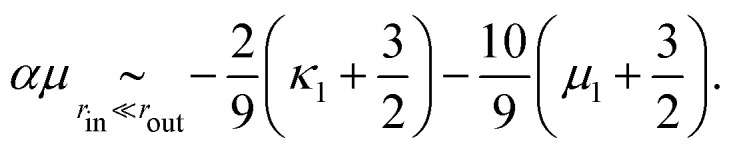


At this order in nonlinearity, stress propagation in a medium with *α* = 0 resembles that in a linear medium (namely 
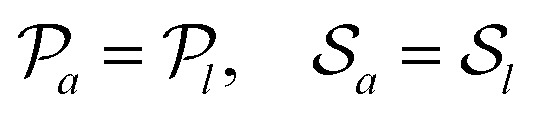
). In contrast, a medium with a negative (positive) *α* harnesses the anisotropy of the active unit to produce an additional medium-wide contraction (expansion). eqn (12) is formally valid for local stresses much smaller than the elastic moduli of the medium (
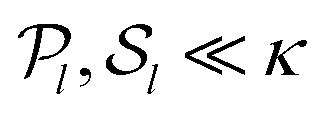
, where “*κ*” stands for the typical magnitude of the linear moduli). It implies that when 
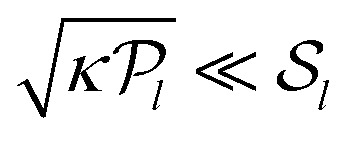
, the sign of the active pressure induced by a highly anisotropic active unit is determined not by the values (
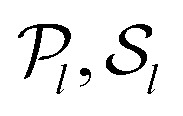
) characterizing the active unit, but by the properties of the medium through the sign of *α*.

**Fig. 2:**
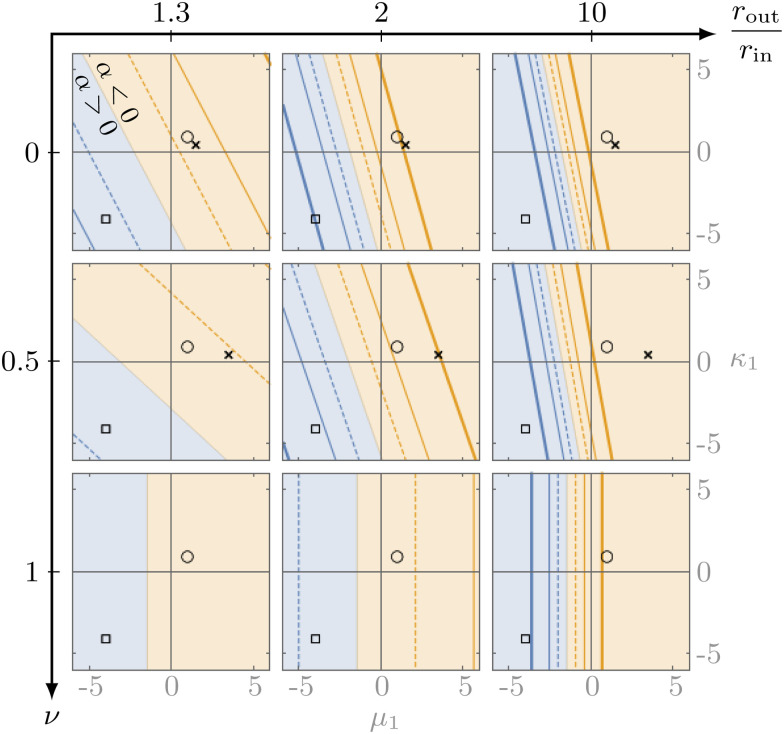
Bucklable materials (*κ*_1_, *μ*_1_ > 0) rectify towards contraction (yellow), while very anti-bucklable materials (*κ*_1_, *μ*_1_ < −3/2) rectify towards expansion (blue). Contour plot of *α*, indicating the overall sign of rectification as a function of the relative system size *r*_out_/*r*_in_, the Poisson ratio *ν* (*ν* = 1 denotes incompressibility in 2D) and the nonlinear corrections to the moduli *κ*_1_, *μ*_1_. The contour lines denote |*α*|*μ* = 2 (thick), 1 (thin), 0.5 (dashed). Crosses indicate constitutively linear materials where only geometrical nonlinearities are present (for *ν* = 1 they are far to the right). As shown in eqn (7), *κ*_1_ and *μ*_1_ encapsulate both geometrical and constitutive (through *κ*′, *μ*′) contributions. As a result, these crosses do not lie at the origin. Circles and squares point out specific media discussed in Fig. 4.

**Fig. 3:**
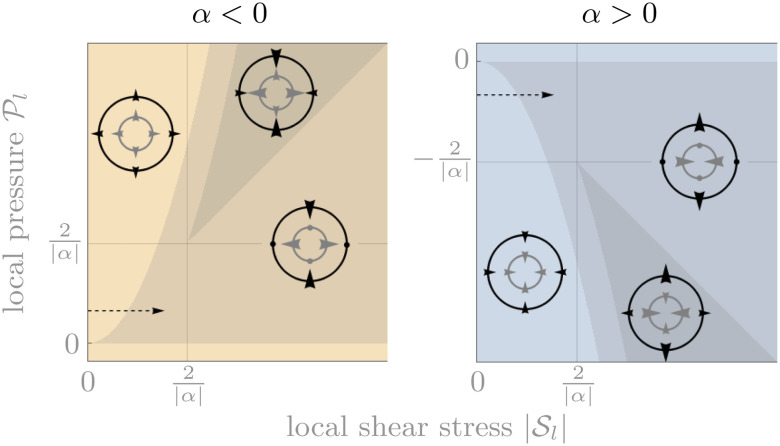
Dependence of the signs of the components of the active stress as functions of the local stress components. Concentric circles illustrate the qualitative rectification situation in each region, with grey (black) arrows indicating directions of the elements of the local (active) stress. Regions without shading correspond to situations where the signs of the active stress components are the same as in the absence of rectification. In regions with intermediate shading (
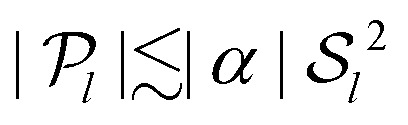
), the sign of 
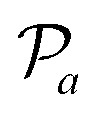
 is reversed. In the dark regions, 
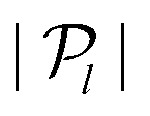
 and 
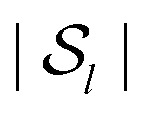
 are so large that all components of ***σ̄***^*a*^ (dark regions) are reversed [ESI†]. These changes of signs are illustrated by arrows in the small pictures. Some arrows are replaced by circles in the intermediate shading regime to indicate that they are smaller than the other arrows and can point either way. Note that changes in the values of the dimensionless parameters *r*_out_/*r*_in_, *κ*/*μ*, *κ*_1_ and *μ*_1_ result in a rescaling of these diagrams through the value of *α*, but leave them otherwise unaffected.

We illustrate the influence of the material's properties on the sign of *α* in Fig. 2, which indicates a clear tendency of fiber-like (granular-like) materials towards contractile (expansile) rectification. Indeed, when *κ*_1_ and *μ*_1_ are both larger (smaller) than a critical value of −3/2, the system always rectifies towards contraction (expansion). As a result, a material with *κ*_1_ = *μ*_1_ = 0 is contractile because of the contractile character of geometrical nonlinearities described by eqn (3). Media with *κ*_1_ > −3/2 but *μ*_1_ < −3/2 or the reverse can be either contractile or expansile depending on the system size *r*_out_/*r*_in_ and Poisson's ratio *ν* = (*κ* − *μ*)/(*κ* + *μ*). Finally, |*α*| increases with increasing *r*_out_ such that |*α*(∞) − *α*(*r*_out_)| ∝ (*r*_in_/*r*_out_)^2^ for large *r*_out_ (see ESI†), implying that larger systems rectify more. For example, fiber networks with a larger *r*_out_ allow for more extensive buckling, resulting in stronger rectification and the coming together of the contour lines of Fig. 2 as *r*_out_ increases. Finally, Fig. 3 shows that for large enough local stresses, rectification can cause a sign-switching not only in the active pressure but in all components of the active stress tensor ***σ̄***^*a*^.

### Simulation of rectification in the presence of finite nonlinearities

While these calculations are strictly valid only for small local stresses, one may hope that eqn (12) remains qualitatively correct for strong active units with 
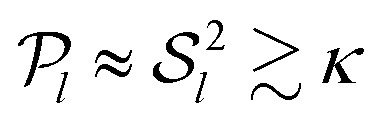
. We test this expectation through finite element simulations [ESI†] of a fully (*i.e.*, not weakly) nonlinear model with an elastic energy density14
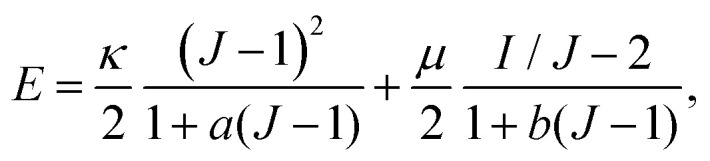
where *J* = det(**1** + ***η***), *I* = Tr(**1** + 2***ε***) and the constants *a*, *b* are defined through *κ*_1_ = 1/2 − 3*a*, *μ*_1_ = −3/2 − *b*. The case *a* = *b* = 0 corresponds to a compressible neo-Hookean model for rubber elasticity.

**Fig. 4:**
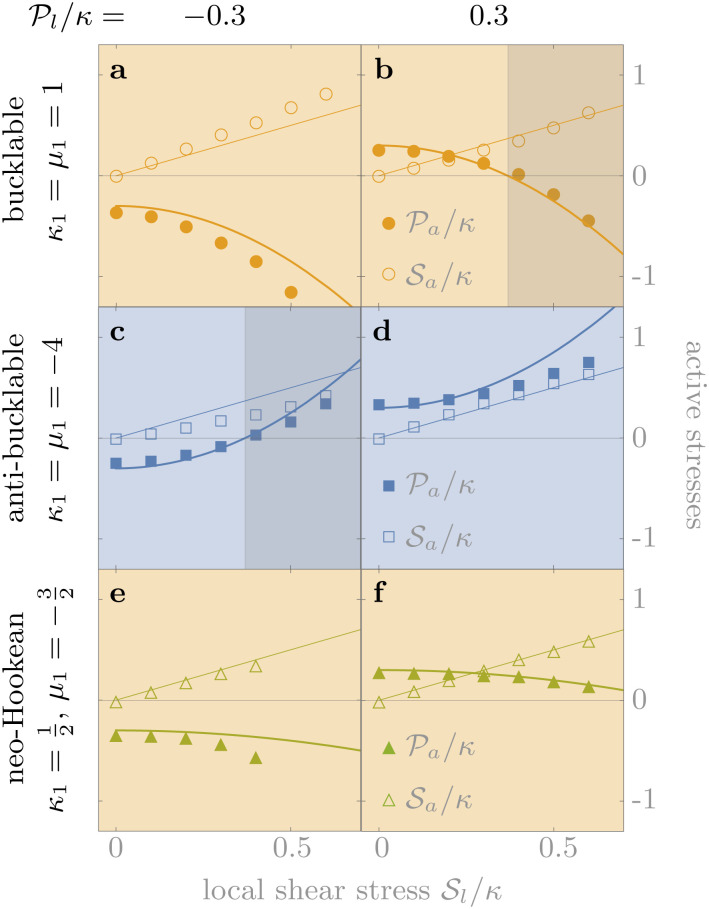
The small-stress asymptotic prediction of eqn (12) (lines) accurately capture the finite-element simulation results (symbols) even for intermediate stress values. Here *ν* = 0.1 and *r*_out_/*r*_in_ = 2 in the geometry of Fig. 1. (a and b) A fiber-like bucklable model, (c and d) a very anti-bucklable model mimicking a granular medium, (e and f) a neo-Hookean model. The values of 
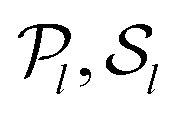
 pictured in (b and c) are marked by dashed arrows in Fig. 3, and background shading follows the same convention. The estimated magnitude of the error induced by the finiteness of the simulation mesh size is comparable to the symbol size.

We illustrate a bucklable and an anti-bucklable material in Fig. 4 by choosing two media with *κ*_1_ = *μ*_1_ = 1 and *κ*_1_ = *μ*_1_ = −4 (equidistant from −3/2, as denoted by symbols in Fig. 2). We also present the rectification obtained for the neo-Hookean model, which corresponds to *κ*_1_ = 1/2, *μ*_1_ = −3/2. As expected, the anti-bucklable material induces expansion, while the other two cause contraction. The quantitative predictions of eqn (12) moreover remain largely valid up to local stress values comparable with the bulk modulus of the network, which implies deformations of the medium of order one. These conclusions also hold in other parameter regimes and for a model specifically designed to mimic the shear-stiffening behavior of fiber networks (Fig. S4, ESI†).^5^ In addition, simulations of isotropic active units with large local stress values suggest that rectification effects also manifest in that case (Fig. S5, ESI†).^9^

## Discussion

Our intuition of the mechanics of nonlinear materials is largely based on deforming their outer boundary. We thus expect a uniformly compressed material to respond with an expansile stress, while applying shear will elicit an opposing shear stress. In this study, we show that if the forces are exerted from the *inside* of the material, these expectations can be upset. In the most extreme cases, an embedded active unit that expands (contracts) in all directions can elicit contractile (expansile) stresses in all directions. The system thus “forgets” the shape of the active units, and its large-scale behavior is controlled by the characteristics of the elastic material instead. Expansion- and shear-stiffening (softening) materials thus always rectify towards contraction (expansion). This rectification tends to be stronger in more compressible materials and in larger systems. These behaviors arise in a continuum model with or without constitutive nonlinearities, and are thus generic in elastic media beyond previously studied discrete fiber networks.

While most of our calculations are conducted in a circular 2D system with a single active unit, they are likely to remain valid in more complex settings provided the elastic medium is homogeneous or displays heterogeneities whose statistics is translationally invariant. Indeed, under these conditions ref. 9, 19 show that if an active unit is far enough away from the boundary of the medium and from other active units, its contribution to the total active stress is independent of the characteristics of either. This remains true as long as the distance between active units is larger than the distance over which each of them induces significant nonlinear deformations. In our small-strain formalism (which also describes intermediate strains well), this distance is of the order of r* ∼ 10*r*_in_ [ESI†]. This result provides a guideline to predict rectification in complex materials, whose elastic characteristics are often length and time scale dependent. For instance, over a time scale of seconds many biopolymer networks are compressible over micrometer length scales, but incompressible over millimeters as the solvent cannot be quickly drained out.^31^ Since rectification is insensitive on the elasticity of the material beyond r*, it only depends the elastic characteristics of the material on this scale, which is of the order of the size *r*_in_ of its active units. Similarly, the relevant time scale is that which the active unit can be expected to exert a force, *e.g.*, the typical detachment time of a myosin minifilament or the time scale over which the protein filaments detach from one another and flow.

In the strongly nonlinear regime, rectification in fiber networks is strikingly similar to the results of our weakly nonlinear formalism,^9^ which may explain why actomyosin networks are always contractile despite the presence of mixed force dipoles.^32,33^ These simulations moreover indicate that rectification in 2D and 3D systems are remarkably similar, suggesting that our results are relevant not only for locally planar contractile systems such as the actin cortex, but also for three-dimensional active materials. Besides, contractility can arise in bucklable fiber networks even in the absence of molecular motors, by rectifying the forces from the active binding and unbinding of crosslinkers when detailed balance is violated.^34^ The application of rectification to discrete granular media and other amorphous solids remains to be investigated. Experiments do however suggest that the elastic response of a foam to a shear transformation zone becomes more isotropic in the vicinity of the jamming transition,^35^ where nonlinear effects are expected to play a large role. We speculate that such effects could be explained by the type of rectification described here. They could then significantly affect the characteristics of the yielding transition in nearly-jammed systems.^36^

## Data availability

The authors declare that the data supporting the findings of this study are available within the paper and its ESI,† or the data are available from the corresponding authors upon request.

## Author contributions

M. L designed the research. F. B. and G. S. carried out the analytical and numerical calculations. The manuscript was written by M. L., F. B., and G. S.

## Conflicts of interest

The authors declare no competing interests.

## Supplementary Material

SM-019-D2SM01606K-s001
