# Supplementary material for: Generic stress rectification in nonlinear elastic media
Source: Soft Matter. 2023 Apr 4;19(16):2970–6. doi: 10.1039/d2sm01606k (PMC10131159; doi:10.1039/d2sm01606k)
Supplement: SM-019-D2SM01606K-s001 [file SM-019-D2SM01606K-s001.pdf]

Electronic Supplementary Material (ESI) for Soft Matter.
This journal is © The Royal Society of Chemistry 2023

Supporting information for "Generic stress rectification in nonlinear elastic media"

I. CONSTITUTIVELY LINEAR MEDIA RECTIFY TOWARDS CONTRACTION

In this section, we consider only geometrical nonlinearities and set out to prove the inequality in Eq. (3). Firstly, the elastic energy density E is written with the Green-Lagrange strain tensor $\boldsymbol{\varepsilon} = (\boldsymbol{\eta} + \boldsymbol{\eta}^{\mathsf{T}} + \boldsymbol{\eta}^{\mathsf{T}}\boldsymbol{\eta})/2$, which depends quadratically on the displacement gradient $\eta_{ij} = \partial u_i/\partial x_j$. We further define the symmetric part of the displacement gradient $U_{ij} = (\eta_{ij} + \eta_{ji})/2$ which corresponds to the linearized strain. The stress tensor which naturally derives from E describes the surface force measured in the initial space with respect to the initial area: $\mathrm{d}\mathbf{F}^0/\mathrm{d}\mathbf{a} = \partial E/\partial \boldsymbol{\varepsilon}$, it is known as the second Piola-Kirchhoff stress. Then in order to find the Cauchy stress measured fully in the target space: $\boldsymbol{\sigma} = \mathrm{d}\mathbf{F}/\mathrm{d}\mathbf{A}$, we need to transform the surface force and the area as

$$\mathrm{d}\mathbf{F} = (\mathbf{1} + \boldsymbol{\eta})\,\mathrm{d}\mathbf{F}^0 \quad \text{and} \quad \mathrm{d}\mathbf{a} = \frac{\mathbf{1} + \boldsymbol{\eta}^{\mathsf{T}}}{\det(\mathbf{1} + \boldsymbol{\eta})}\mathrm{d}\mathbf{A}, \tag{S1}$$

which ultimately gives the formula for the Cauchy stress in the main text [1]. Then, given the quadratic energy density $E = \kappa\varepsilon_{ii}^2/2 + \mu(\varepsilon_{ij}^2 - \epsilon_{ii}^2/d)$, the stress-strain relation displays a linear stress term $\boldsymbol{\sigma}^L$ proportional to $\mathbf{U}$, and a term which includes the geometrical nonlinearities $\boldsymbol{\sigma}^G$:

$$\boldsymbol{\sigma}^L = \big(\kappa - 2\mu/d\big)U_{ii}\,\mathbf{1} + 2\mu\,\mathbf{U}, \tag{S2a}$$

$$\boldsymbol{\sigma}^G = \big(\kappa - 2\mu/d\big)\big(\eta_{ij}^2/2 - U_{ii}^2\big)\mathbf{1} + \mu\big(4\mathbf{U}^2 + \boldsymbol{\eta}\boldsymbol{\eta}^{\mathsf{T}}\big) + 2\big(\kappa - 2\mu/d - \mu\big)U_{ii}\mathbf{U} + \mathcal{O}\big(\eta^3\big). \tag{S2b}$$

Secondly, given the force balance equation $f_i = -\partial\sigma_{ij}/\partial X_j$, the difference between the local and active coarse-grained stresses can be integrated by part to read

$$\bar{\boldsymbol{\sigma}}^a - \bar{\boldsymbol{\sigma}}^l = \frac{1}{V}\int_\Omega \boldsymbol{\sigma}\,\mathrm{d}V = \frac{1}{V}\int_\Omega \boldsymbol{\sigma}\det(\mathbf{1} + \boldsymbol{\eta})\,\mathrm{d}v, \tag{S3}$$

where $\mathrm{d}v$ is the volume element in the initial space. Equation (S3) is known as the mean stress theorem [2, 3]. Due to this relation, writing $\mathbf{S} = \boldsymbol{\sigma}\det(\mathbf{1} + \boldsymbol{\eta})$ and decomposing $\mathbf{S} = \mathbf{S}^L + \mathbf{S}^G$ as we did $\boldsymbol{\sigma}$ in Eq. (S2), the pressure difference reads $P_a - P_l = -\int S_{ii}/(Vd)$. Due to our fixed boundary condition, the integral of the trace of the linear term $\mathbf{S}^L = \boldsymbol{\sigma}^L$ vanishes and the trace of the nonlinear term is expressed in a closed form as

$$S_{ii}^G = \frac{\kappa}{2}\left(d\eta_{ij}^2 + 4\varepsilon_{ii}^2\right) + 4\mu\big(\varepsilon_{ij}^2 - \varepsilon_{ii}^2/d\big). \tag{S4}$$

Here, κ and μ are both positive for mechanical stability. Thus, for $d \geq 2$, the geometrical term S_{ii}^G always gives a positive (contractile) contribution to the active stress. Indeed, using the eigenvalues $\lambda_i \in \mathbb{R}$ of the symmetric matrix $\boldsymbol{\varepsilon}$, we can rewrite $\varepsilon_{ij}^2 - \varepsilon_{ii}^2/d = \sum_{i<j}(\lambda_i - \lambda_j)^2/d$, which is always non-negative. As a result S_{ii}^G is a sum of squares that is also non-negative, implying the inequality of Eq. (3): $P_a - P_l \leqslant 0$.

Finally, we present an alternative derivation of this relation that highlights its frame indifference. The integrand of Eq. (3) can be rewritten using the deformation gradient $\boldsymbol{\Lambda} = \mathbf{1} + \boldsymbol{\eta}$ and the right Cauchy-Green deformation tensor $\mathbf{C} = \boldsymbol{\Lambda}^{\mathsf{T}}\boldsymbol{\Lambda} = \mathbf{1} + 2\boldsymbol{\varepsilon}$. Indeed, since

$$\mathbf{S} = \boldsymbol{\Lambda}\frac{\partial E}{\partial \boldsymbol{\varepsilon}}\boldsymbol{\Lambda}^{\mathsf{T}}, \quad \text{where} \quad \frac{\partial E}{\partial \boldsymbol{\varepsilon}} = \frac{\kappa}{2}(C_{ii} - d)\mathbf{1} + \mu\big(\mathbf{C} - C_{ii}\mathbf{1}/d\big), \tag{S5}$$

we can write

$$S_{ii} = \frac{\kappa}{2}\left[(C_{ii} - d)^2 + dC_{ii}\right] + \mu\big[C_{ij}^2 - C_{ii}^2/d\big]. \tag{S6}$$

We see that the right-hand side Eq. (S6) is a sum of squares plus a term $\propto C_{ii} = d + 4\eta_{ii} + 4\eta_{ij}^2$. Since η_{ii} is integrated to zero due to the fixed boundary condition, the term in Eq. (S6) also gives a contractile contribution to $P_a - P_l$.

II. ELASTIC MODULI IN GRANULAR MEDIA NEAR JAMMING

Here, we derive typical values of κ_1 and μ_1 for granular media near the jamming transition. Let us consider a large equilibrium packing of bidisperse frictionless spherical grains in a 2D box with volume fraction ϕ. The grains interact through a harmonic potential $\mathcal{V} \sim k\,\delta^2$, where k is a spring constant, and δ the overlap divided by the sum of the two bead diameters, see Fig. S1. For volume fractions slightly above the jamming transition $\phi_c \approx 0.84$, granular media display a strongly nonlinear elastic behavior. Indeed, multiple simulations and experiments [4, 5] have shown that while the bulk modulus goes to a finite limit in ϕ_c^+ and can thus be approximated by a constant, the shear modulus scales with $\phi - \phi_c$ and vanishes at the transition. This can be expressed as

$$K/k \sim K_0 \quad \text{and} \quad G/k \sim G_0(\phi-\phi_c)^p, \quad \text{for} \quad 0 < \phi - \phi_c \ll 1, \tag{S7}$$

where $K_0, G_0 \approx 0.2$ and $p \approx 0.5$. Based on this model, we impose an isotropic compression characterized by a displacement gradient tensor $\eta_{ij} = -\eta_0\delta_{ij}/d$ on our granular material initially at ϕ_c, where $0 < \eta_0 \ll 1$. This results in a new volume fraction $\phi_0 \sim \phi_c(1+\eta_0)$. We then compute the elastic moduli κ and μ, and their nonlinear corrections κ_1 and μ_1 around this value of ϕ_0. Let the bulk strain $\eta_{ii} = -\eta_0 + \delta\eta$, where $|\delta\eta| \ll \eta_0$, corresponding to a volume fraction $\phi \sim \phi_0 - \phi_c\delta\eta$. Then similarly to Eq. (6), the moduli are expressed as $K \sim \kappa(1+\kappa_1\delta\eta)$ and $G \sim \mu(1+\mu_1\delta\eta)$, where the parameters are derived from Eq. (S7):

$$\begin{aligned} &\kappa/k \sim K_0, && \kappa_1 = 0, \\ &\mu/k \sim G_0(\phi_0-\phi_c)^p, && \mu_1 \sim -\frac{p\,\phi_c}{\phi_0-\phi_c}. \end{aligned} \tag{S8}$$

Therefore, while κ_1 vanishes, μ_1 diverges at the transition and scales as $(\phi_0-\phi_c)^{-1}$. And close to the transition, around *e.g.* $\phi_0 - \phi_c = 0.001$, 0.01 or 0.1, we find respectively $\mu_1 \approx -400$, -40 or -4 as in the main text.

III. COARSE-GRAINED STRESSES IN THE CIRCULAR GEOMETRY

In this section, we present the analytical calculations leading to the expressions of the coarse-grained stresses $\bar{\boldsymbol{\sigma}}$ in Eq. (9) and of the rectification coefficient α of Eq. (10) of the main text. We first rewrite the coarse-grained stresses in the initial space where the calculations will be easier to handle in Sec. III A. Then in Sec. III B we present the Ansatz for the displacement field that allows us to solve the force balance condition. In Sec. III C, we show the expressions of the coarse-grained pressures and shear stresses with the stress and displacement fields. We finally display the detailed expressions of the coarse-grained stresses $\bar{\boldsymbol{\sigma}}$ in the circular geometry as well as the expression of α in Sec. III D.

A. Rewriting the coarse-grained stresses in the initial space

In order to calculate the coarse-grained stresses, we need to express them in the initial configuration, where the force density $\boldsymbol{\phi} = \mathbf{f}/\det(\mathbf{1}+\boldsymbol{\eta})$ is related to the first Piola-Kirchhoff stress tensor $\boldsymbol{\tau} = (\mathbf{1}+\boldsymbol{\eta})\frac{\partial E}{\partial \boldsymbol{\varepsilon}}$. The force balance equation thus reads $\phi_i = -\partial_j\tau_{ij}$ and the coarse-grained stresses can be rewritten as

$$\bar{\sigma}^a_{ij} = \frac{1}{V}\oint_{\partial\Omega} \tau_{ik}X_j\,\mathrm{d}a_k \quad \text{and} \quad \bar{\sigma}^l_{ij} = -\frac{1}{V}\int_{\Omega}\phi_i X_j\,\mathrm{d}v, \tag{S9}$$

where $\mathrm{d}\mathbf{a}$ is the outward-directed area element in the initial space. As before in Sec. I, we distinguish the linear term $\boldsymbol{\tau}^L$ (which is the same in the initial and target spaces $\boldsymbol{\tau}^L = \boldsymbol{\sigma}^L$), from the nonlinear term $\boldsymbol{\tau}^{\mathrm{NL}} = \boldsymbol{\tau}^G + \boldsymbol{\tau}^C$. This

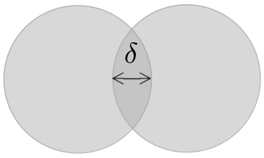

FIG. S1. Overlap between two interacting beads in a granular simulation.

last equality distinguishes the geometrical and constitutive nonlinearities. Given the non-harmonic energy density of Eq. (4), up to second order, the terms read

$$\boldsymbol{\tau}^L = \big(\kappa - 2\mu/d\big)U_{ii}\,\mathbf{1} + 2\mu\,\mathbf{U}, \tag{S10a}$$

$$\boldsymbol{\tau}^G = \big(\kappa - 2\mu/d\big)\eta_{ij}^2\,\mathbf{1}/2 + \mu\,\boldsymbol{\eta}^\mathsf{T}\boldsymbol{\eta} + \big(\kappa - 2\mu/d\big)U_{ii}\,\boldsymbol{\eta} + 2\mu\,\boldsymbol{\eta}\mathbf{U} + \mathcal{O}\big(\eta^3\big). \tag{S10b}$$

$$\boldsymbol{\tau}^C = 3\big(\kappa' - 2\mu'/d\big)U_{ii}^2\,\mathbf{1}/2 + \mu' U_{ij}^2\,\mathbf{1} + 2\mu' U_{ii}\,\mathbf{U} + \mathcal{O}\big(\eta^3\big). \tag{S10c}$$

B. Ansatz for the displacement field

In the initial space, the material is subjected to zero body force except at $r_{\rm in}$, where the stress is discontinuous. The fixed boundary at $r_{\rm out}$ and the imposed displacement at $r_{\rm in}$ additionally impose boundary conditions on the stress and displacement fields, resulting in the following system of equations:

$$\begin{cases} \boldsymbol{\nabla}\cdot\boldsymbol{\tau}^\mathsf{T} = \mathbf{0}, & \text{for } r \in [0, r_{\rm in}) \cup (r_{\rm in}, r_{\rm out}) \\ \mathbf{u} = \mathbf{0}, & \text{at } r = 0 \text{ and } r = r_{\rm out} \\ \mathbf{u} = r_{\rm in}\left[e_0 + e_2\cos(2\theta)\right]\hat{\mathbf{r}}, & \text{at } r = r_{\rm in} \end{cases}. \tag{S11}$$

We solve this system perturbatively by expanding $\mathbf{u}, \boldsymbol{\eta}, \boldsymbol{\tau}$ in the small scalar quantity

$$\eta \sim |e_0| + |e_2|. \tag{S12}$$

The displacement gradient is hence written $\boldsymbol{\eta} = \boldsymbol{\eta}^L + \boldsymbol{\eta}^{\rm NL} + \mathcal{O}\big(\eta^3\big)$ where the L and NL superscript refer to linear and quadratic (nonlinear) terms in η. This allows us to write the stress tensor as

$$\boldsymbol{\tau} = \boldsymbol{\tau}^L\big(\boldsymbol{\eta}^L\big) + \boldsymbol{\tau}^L\big(\boldsymbol{\eta}^{\rm NL}\big) + \boldsymbol{\tau}^{\rm NL}\big(\boldsymbol{\eta}^L\big) + \mathcal{O}\big(\eta^3\big), \tag{S13}$$

where $\boldsymbol{\tau}^L\big(\boldsymbol{\eta}^L\big)$ is of order 1, while the next two are of order 2. The linear displacement field $\mathbf{u}^L$ is the solution of $\partial_i\tau_{ji}^L(\boldsymbol{\eta}^L) = 0$. This is solved by decomposing $\mathbf{u}^L$ in the following Fourier modes due to the form of the imposed displacement:

$$u_r^L/r_{\rm in} = e_0\zeta_0(r) + e_2\zeta_2(r)\cos 2\theta, \tag{S14a}$$

$$u_\theta^L/r_{\rm in} = e_2\omega_2(r)\sin 2\theta. \tag{S14b}$$

Here $\zeta_0(r)$, $\zeta_2(r)$, $\omega_2(r)$ are sums of r^k, with $k \in \{-3, -1, 1, 3\}$ and coefficients depending on the boundary conditions. Then, at the first nonlinear order, $\mathbf{u}^{\rm NL}$ is the solution of the linear equation $\partial_i\tau_{ji}^L(\boldsymbol{\eta}^{\rm NL}) = -\partial_i\tau_{ji}^{\rm NL}(\boldsymbol{\eta}^L)$ which is solved by expanding $\mathbf{u}^{\rm NL}$ as

$$u_r^{\rm NL}/r_{\rm in} = e_2^2\xi_0(r) + e_0e_2\xi_2(r)\cos 2\theta + e_2^2\xi_4(r)\cos 4\theta, \tag{S15a}$$

$$u_\theta^{\rm NL}/r_{\rm in} = e_0e_2\pi_2(r)\sin 2\theta + e_2^2\pi_4(r)\sin 4\theta, \tag{S15b}$$

where the $\xi_i(r)$, $\pi_i(r)$ are again sums of r^k with k odd between -7 and $+5$. As a consequence, we obtain in Sec. III D the strain and stress fields up to second order in η.

C. Calculations of the coarse-grained stresses

We compute the coarse-grained active stress $\bar{\boldsymbol{\sigma}}^a$ and local stress $\bar{\boldsymbol{\sigma}}^l$ in the circular geometry by integrating the stresses in the material as in Eq. (S9). The coarse-grained stresses are expressed in Cartesian coordinates, while the stress field is more easily expressed in polar coordinates. In the following (and in this subsection only), we denote Cartesian indices x, y with Greek letters (μ, ν) and polar indices r, θ with Latin letters (i, j, k). The change-of-basis matrix between these two systems reads $\mathbf{R} = \left(\begin{smallmatrix}\cos\theta & -\sin\theta \\ \sin\theta & \cos\theta\end{smallmatrix}\right)$. In the circular geometry of Fig. 1 where the active unit

produces a discontinuity in the stress at $r_{\rm in}$ in the initial configuration, the coarse-grained stresses are expressed as follows:

$$\bar{\sigma}^a_{\mu\nu} = \frac{1}{\pi r_{\rm out}^2}\oint_{\partial\Omega} R_{\mu i}\,\tau_{ik}\,R_{\nu j}\,X_j\,{\rm d}a_k = \frac{1}{\pi}\int_0^{2\pi}{\rm d}\theta\,R_{\mu i}\,\tau_{ir}(r_{\rm out},\theta)R_{\nu r}, \tag{S16a}$$

$$\bar{\sigma}^l_{\mu\nu} = \frac{1}{\pi r_{\rm out}^2}\int_{\Omega} R_{\mu i}\,\partial_k\tau_{ik}\,R_{\nu j}\,X_j\,{\rm d}V = \frac{1}{\pi}\frac{r_{\rm in}^2}{r_{\rm out}^2}\int_0^{2\pi}{\rm d}\theta\,R_{\mu i}\Big[\tau_{ir}(r_{\rm in}^+,\theta)-\tau_{ir}(r_{\rm in}^-,\theta)\Big]R_{\nu r}\left[1+\frac{u_r(r_{\rm in},\theta)}{r_{\rm in}}\right], \tag{S16b}$$

where $\partial_k\tau_{ik}$ denotes the stress divergence expressed in polar coordinates. Then, introducing $\beta = (r_{\rm out}/r_{\rm in})^2$, the active pressure and shear stress rescaled so as to compensate for dilution read

$$\beta(\bar{\sigma}^a_{xx}+\bar{\sigma}^a_{yy}) = -2\mathcal{P}_a = \frac{\beta}{\pi}\int \tau_{rr}(r_{\rm out},\theta), \tag{S17a}$$

$$\beta(\bar{\sigma}^a_{xx}-\bar{\sigma}^a_{yy}) = -2\mathcal{S}_a = \frac{\beta}{\pi}\int \Big[\tau_{rr}(r_{\rm out},\theta)\cos 2\theta - \tau_{\theta r}(r_{\rm out},\theta)\sin 2\theta\Big], \tag{S17b}$$

with similar expressions for the local pressure and shear stress:

$$-2\mathcal{P}_l = \frac{1}{\pi}\int\Big[\tau_{rr}(r_{\rm in}^+,\theta)-\tau_{rr}(r_{\rm in}^-,\theta)\Big]\left[1+\frac{u_r(r_{\rm in},\theta)}{r_{\rm in}}\right], \tag{S18a}$$

$$-2\mathcal{S}_l = \frac{1}{\pi}\int\left\{\Big[\tau_{rr}(r_{\rm in}^+,\theta)-\tau_{rr}(r_{\rm in}^-,\theta)\Big]\cos 2\theta - \Big[\tau_{\theta r}(r_{\rm in}^+,\theta)-\tau_{\theta r}(r_{\rm in}^-,\theta)\Big]\sin 2\theta\right\}\left[1+\frac{u_r(r_{\rm in},\theta)}{r_{\rm in}}\right]. \tag{S18b}$$

D. Full expressions of the coarse-grained stresses

In Eqs. (9-10), we consider small displacements with two independent parameters $e_0, e_2 \ll 1$. In the weakly nonlinear formalism, the simplest possible rectification requires that the term in e_0 be similar (and of opposite sign) to the term in e_2^2. In this regime, given $\epsilon \ll 1$, the displacement parameters read $e_0 = \epsilon\,\tilde{e}_0$ and $e_2 = \sqrt{\epsilon}\,\tilde{e}_2$. As a result $\mathcal{P}_x = \epsilon\tilde{\mathcal{P}}_x$ and $\mathcal{S}_x = \sqrt{\epsilon}\tilde{\mathcal{S}}_x$ for $x \in \{l, a\}$, where the tildes denote quantities of order one. Then to lowest order in ϵ, Eq. (9) can be rewritten as

$$\tilde{\mathcal{P}}_x = A_x\tilde{e}_0 + B_x\tilde{e}_2^2 + \mathcal{O}(\epsilon), \tag{S19a}$$

$$\tilde{\mathcal{S}}_x = C_x\tilde{e}_2 + \mathcal{O}(\epsilon), \tag{S19b}$$

implying that the "$\sim$" symbols of Eq. (10) denote equalities to lowest order in ϵ. The rectification behavior thus depends on the ratio $\tilde{\mathcal{P}}_l/(\alpha\tilde{\mathcal{S}}_l^2)$, *i.e.* on $\tilde{e}_0/\tilde{e}_2^2$. We further display the complete expressions of the coefficients in Eq. (9) obtained after finding the strain field through force balance (see Sec. III B) and integrating the resulting stress via Eq. (S10), as in (S17) and (S18). In order to make sense of the cumbersome expressions of the A_x, B_x and C_x, we introduce several quantities: $X = (3-\nu)^2(1+\beta^2) + 2(3-6\nu-\nu^2)\beta$,

$$\begin{aligned} a_0 = {} & 1215 - 1863\nu + 756\nu^2 - 126\nu^3 + 87\nu^4 - 43\nu^5 + 6\nu^6 \\ & + (81 - 2457\nu + 2412\nu^2 - 594\nu^3 + 265\nu^4 + 155\nu^5 - 22\nu^6)\beta \\ & + (1782 - 3798\nu + 6216\nu^2 - 3948\nu^3 + 326\nu^4 - 286\nu^5 + 28\nu^6)\beta^2 \\ & - (918 + 1170\nu - 2616\nu^2 + 1044\nu^3 + 54\nu^4 - 262\nu^5 + 12\nu^6)\beta^3 \\ & + (459 + 45\nu + 132\nu^2 - 918\nu^3 + 515\nu^4 - 71\nu^5 - 2\nu^6)\beta^4 \\ & - (891 - 1755\nu + 1188\nu^2 - 294\nu^3 - 13\nu^4 + 17\nu^5 - 2\nu^6)\beta^5, \end{aligned}$$

$$\begin{aligned} a_1 = {} & 1863 - 756\nu - 54\nu^2 + 12\nu^3 + 7\nu^4 + (1377 - 1260\nu + 102\nu^2 - 108\nu^3 - 31\nu^4)\beta \\ & + (1782 - 1368\nu + 804\nu^2 + 168\nu^3 + 54\nu^4)\beta^2 + (162 - 504\nu - 132\nu^2 - 24\nu^3 - 46\nu^4)\beta^3 \\ & + (243 + 108\nu + 18\nu^2 - 84\nu^3 + 19\nu^4)\beta^4 - (243 - 324\nu + 162\nu^2 - 36\nu^3 + 3\nu^4)\beta^5, \end{aligned}$$

$$
\begin{aligned}
a_2 = {} & 2511 - 4104\nu + 4023\nu^2 - 1416\nu^3 + 173\nu^4 - 48\nu^5 + 13\nu^6 \\
& + (1377 - 8352\nu + 6369\nu^2 - 2568\nu^3 - 77\nu^4 + 104\nu^5 - 53\nu^6)\beta \\
& + (6966 - 16128\nu + 17430\nu^2 - 6480\nu^3 + 1890\nu^4 - 176\nu^5 + 82\nu^6)\beta^2 \\
& + (2754 - 9216\nu + 6162\nu^2 - 1392\nu^3 - 794\nu^4 + 240\nu^5 - 58\nu^6)\beta^3 \\
& + (2187 - 3672\nu + 3843\nu^2 - 1896\nu^3 + 545\nu^4 - 128\nu^5 + 17\nu^6)\beta^4 \\
& - (243 - 189\nu^2 + 72\nu^3 + 9\nu^4 - 8\nu^5 + \nu^6)\beta^5,
\end{aligned}
$$

and

$$
\begin{aligned}
b_0 = {} & 81 - 270\nu + 234\nu^2 - 36\nu^3 + 13\nu^4 - 6\nu^5 + (135 - 378\nu + 366\nu^2 - 196\nu^3 - 29\nu^4 + 22\nu^5)\beta \\
& + (162 - 708\nu + 1116\nu^2 - 464\nu^3 + 82\nu^4 - 28\nu^5)\beta^2 + (126 - 564\nu + 540\nu^2 - 168\nu^3 - 106\nu^4 + 12\nu^5)\beta^3 \\
& + (45 - 222\nu + 426\nu^2 - 188\nu^3 + 17\nu^4 + 2\nu^5)\beta^4 + (27 - 162\nu + 198\nu^2 - 100\nu^3 + 23\nu^4 - 2\nu^5)\beta^5,
\end{aligned}
$$

$$
\begin{aligned}
b_1 = {} & 27 - 18\nu + 3\nu^2 + (189 + 18\nu - 11\nu^2)\beta + (174 + 12\nu + 14\nu^2)\beta^2 \\
& + (138 - 12\nu - 6\nu^2)\beta^3 + (39 + 6\nu - \nu^2)\beta^4 + (9 - 6\nu + \nu^2)\beta^5,
\end{aligned}
$$

$$
\begin{aligned}
b_2 = {} & 243 - 270\nu + 84\nu^2 - 34\nu^3 + 9\nu^4 + (189 - 522\nu + 404\nu^2 + 58\nu^3 - 33\nu^4)\beta \\
& + (510 - 1356\nu + 568\nu^2 - 84\nu^3 + 42\nu^4)\beta^2 + (474 - 612\nu + 312\nu^2 + 100\nu^3 - 18\nu^4)\beta^3 \\
& + (159 - 486\nu + 244\nu^2 - 10\nu^3 - 3\nu^4)\beta^4 + (153 - 210\nu + 116\nu^2 - 30\nu^3 + 3\nu^4)\beta^5.
\end{aligned}
$$

In the end, we find

$$A_l = A_a = \frac{4\kappa\beta}{(1+\nu)(\beta-1)}, \tag{S20a}$$

$$B_l = -\kappa\beta\frac{(3-\nu)a_0 + (1-\nu)^2(1+\nu)a_1\,\kappa_1 + (1-\nu)a_2\,\mu_1}{(3-\nu)^2(1+\nu)(\beta-1)^2X^2}, \tag{S20b}$$

$$B_a = -\kappa\beta\frac{b_0 + (1-\nu)^2(1+\nu)b_1\,\kappa_1 + (1-\nu)b_2\,\mu_1}{(1+\nu)(\beta-1)^2X^2}, \tag{S20c}$$

$$C_l = C_a = 4\mu\beta\frac{2(3+\nu) + (3-\nu)(\beta+\beta^2)}{(\beta-1)X}. \tag{S20d}$$

As expected from the linear elasticity analysis of Sec. I, the coefficients in front of the linear e_0 and e_2 terms are identical for the local and boundary stresses, but discrepancies appear in the e_2^2 terms. This leads us to define $\alpha = (B_a - B_l)/C_l^2$.

IV. BEHAVIOR OF α AND RECTIFICATION SATURATION RADIUS r^*

To help better understand the lengthy expression of the rectification coefficient $\alpha = (B_a - B_l)/C_l^2$ in subsection III.D, we hereby discuss its dependence on the system size r_{out}. As is apparent from Fig. 2(a), α increases with increasing r_{out} for relatively small systems, then saturates as the size of the system goes to infinity. Indeed, away from the high-stress region close to the active unit, the stress decrease causes the nonlinearities to become negligible in front of the linear terms. Therefore, we examine the radius r^* at which stress propagation switches from nonlinear to linear. To this end we define the system size parameter $\beta = (r_{\text{out}}/r_{\text{in}})^2$. In the limit $\beta \to \infty$, Eq. (S20) leads to $\alpha(\beta) = \alpha_\infty + \alpha_1/\beta + \mathcal{O}(\beta^{-2})$, where

$$
\begin{aligned}
\mu\alpha_\infty &= -\frac{\left(\kappa_1 + \frac{3}{2}\right)(1-\nu^2) + \left(\mu_1 + \frac{3}{2}\right)(5 - 2\nu + \nu^2)}{4}, \\
\mu\alpha_1 &= \frac{\left(\kappa_1 + \frac{3}{2}\right)(15 - 6\nu - 8\nu^2 + 6\nu^3 - 7\nu^4) + \left(\mu_1 + \frac{3}{2}\right)(111 - 36\nu + 50\nu^2 - 20\nu^3 + 7\nu^4)}{4(3-\nu)^2}.
\end{aligned} \tag{S21}
$$

We introduce the value of the parameter β such that α is within 10% of its large-size limit through $\left|\frac{\alpha(\beta_{10}) - \alpha_\infty}{\alpha_\infty}\right| = 0.1$. The square-root of β_{10}, corresponding to the ratio of the radii, lies between 4 and 9, except for insignificant values

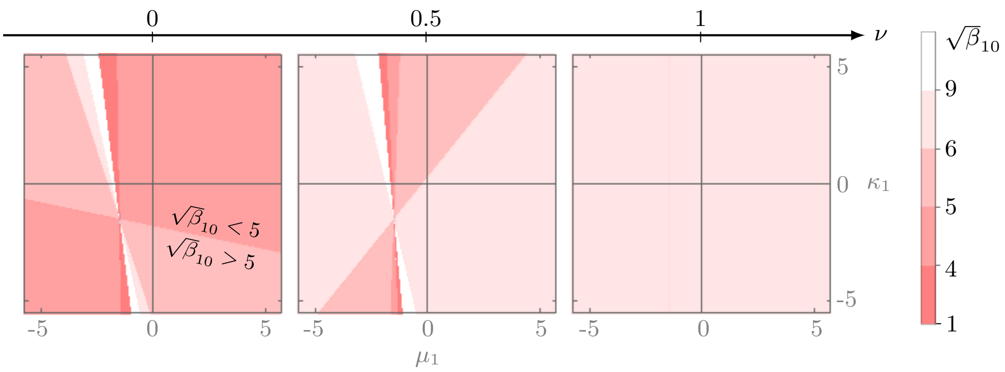

FIG. S2. The rectification saturation radius r^* is generally of the order of r_{in}. Contour plot of $\sqrt{\beta}_{10} = r^*/r_{\text{in}}$ when κ_1 and μ_1 are varied for several values of Poisson's ratio ν. Except in the small white regions, $\sqrt{\beta}_{10} < 9$. In these white regions, $|\alpha|$ tends to take negligible values.

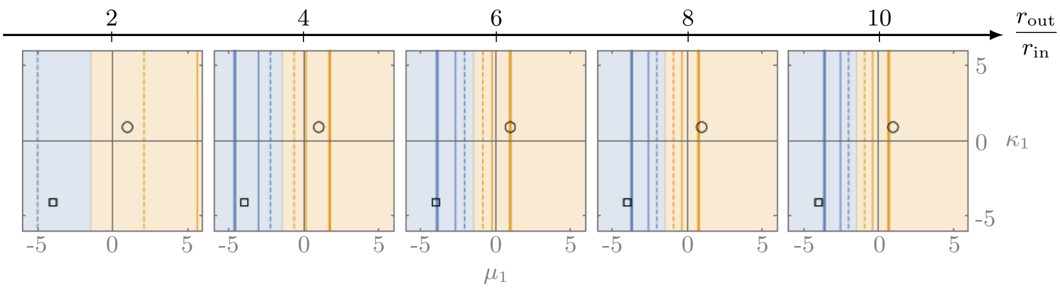

FIG. S3. Rectification happens mostly near the active unit. Additional plots to Fig. 2(a) showing the stabilization of the graphs as the system size increases for a Poisson's ratio $\nu = 1$. The blue and yellow lines at constant $|\alpha|\mu$ stay quite still between $r_{\text{out}}/r_{\text{in}} = 8$ and 10. The behavior is similar for $\nu = 0$.

of α, see Fig. S2. Fig. S3 also illustrates this behavior by showing the stabilization of the lines at constant α as the system size increase. Therefore, defining the rectification saturation radius r^* such that $\beta_{10} = (r^*/r_{\text{in}})^2$, increasing r_{out} past $r^* \sim 10 r_{\text{in}}$ has little influence on the value of $\mathcal{P}_a - \mathcal{P}_l$, *i.e.* on the rectification effect. This indicates that the propagation is nonlinear only up to r^*. In the study of stress propagation from multiple active units, one thus needs to compare this r^* to the typical spacing between two active units.

V. THE RECTIFICATION DIAGRAM

Let us give further explanation to the shadings of the rectification diagram of Fig. 2(b), corresponding to different rectification regimes. In the circular geometry of Fig. 1, provided that α and $\mathcal{P}_l$ have different signs, a change of sign of P_a due to rectification can appear for all values of the local pressure $\mathcal{P}_l$ as long as the local shear stress $|\mathcal{S}_l|$ is large enough. Indeed, in Eq. (10) the sign switching of the active pressure $\mathcal{P}_a$ (*e.g.* $\mathcal{P}_a < 0$ while $\mathcal{P}_l > 0$) requires

$$|\alpha|\mathcal{S}_l^2 \gtrsim |\mathcal{P}_l|. \tag{S22}$$

This sets the boundary between the regions with light shading and the regions with intermediate shading. Then, the extreme case where all active stress components switch sign (*e.g.* $\mathcal{P}_a \pm \mathcal{S}_a < 0$ while $\mathcal{P}_l \pm \mathcal{S}_l > 0$) happens for

$$|\mathcal{S}_l| \lesssim |\mathcal{P}_l| \lesssim |\alpha|\mathcal{S}_l^2 - |\mathcal{S}_l|, \tag{S23}$$

i.e. for $|\mathcal{P}_l|$ and $|\mathcal{S}_l|$ both larger than $2/|\alpha|$. This extreme case corresponds to the dark regions of Fig. 2(b).

VI. FINITE-ELEMENT SIMULATIONS

This section provides further details on the finite element simulations used to produce Fig. 3 of the main text. Sec. VI A describes our simulation methods. In Sec. VI B, we discuss rectification in two additional fully nonlinear models.

A. Methods

We solve the set of equations (S11) via simulations with the finite element software Fenics [6] version 2019.2.0.dev0. We use a mesh with maximal size $l = 0.01$ for $r_{\rm in} = 1$ and $r_{\rm out} = 2$, and another one with $l = 0.1$ for $r_{\rm out} = 10$. They were both created with Gmsh version 4.4.1. In all figures, the error bars correspond to the differences between two meshes at l and $l/10$, which gives roughly 5% of $\mathcal{S}_l$ or $\mathcal{P}_l$ for all points. The meshes are created without enforcing rotational symmetry, which results in small non-zero values for the non-diagonal coefficients that should be zero in a continuum system (*e.g.*, $\bar{\sigma}^l_{xy}$), as shown in Eq. (8). However, we find that these values are smaller than 5% of the diagonal coefficients in all simulations. In the geometry of Fig. 1, if we apply too large a deformation at $r_{\rm in}$, we come into contact with the fixed boundary at $r_{\rm out}$, which poses some numerical issues. Therefore, we can only perform accurate simulations up to about $\eta = |e_0| + |e_2| \sim 0.6$.

B. Additional data

In the main text, we studied two models with clear buckling and anti-buckling behaviors, which lead to a readily observable reversal of the active pressure sign, due to rectification. We also studied a standard neo-Hookean model of rubber in which this behavior is less pronounced. Here, we study another model which can mimic the shear-stiffening behavior of fiber networks. Consistent with analytical predictions, this system displays a smaller propensity for rectification, and we show that the predictions of Eq. (10) remain valid up to intermediate stress values.

In the fully nonlinear model with the elastic energy density of Eq. (11), we introduced the parameters a, b such that $\kappa_1 = 1/2 - 3a$ and $\mu_1 = -3/2 - b$. In Fig. 3, we showed the good agreement between the weakly nonlinear predictions of Eq. (10) and finite-element simulations for the bucklable and anti-bucklable models obtained by setting $(a, b) = (-1/6, -5/2)$ and $(3/2, 5/2)$ to obtain $(\kappa_1, \mu_1) = (1, 1)$ and $(-4, -4)$ respectively. As well as for the neo-Hookean model, obtained by setting $a = b = 0$ in Eq. (11), which has $\kappa_1 = 1/2$, $\mu_1 = -3/2$. In Fig. S4(a), we show thqt the agreement between the simulations with the neo-Hookean model and Eq. (10) remains quantitative in the

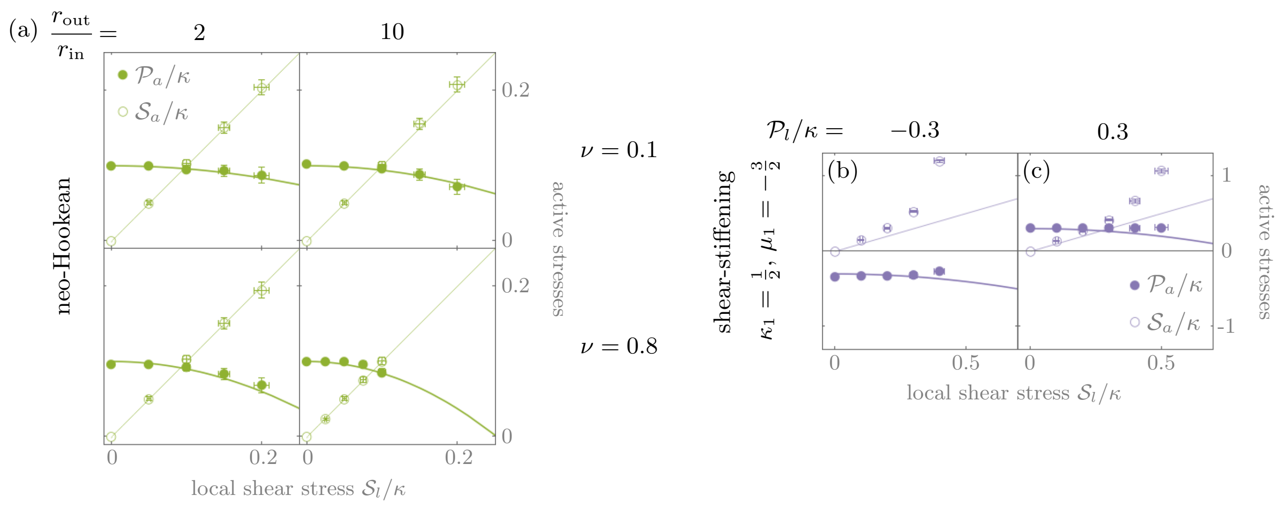

FIG. S4. Additional plots of the coarse-grained stresses are in agreement with Eq. (10) up to intermediate stresses and in a large scale of parameters. (a) Rubber-like neo-Hookean model [$a = b = 0$ in Eq. (11)]. For $\mathcal{P}_l = 0.1\kappa$, the predictions at $\nu = 0.8$ and $r_{\rm out}/r_{\rm in} = 10$ remain quantitatively accurate. (b-c) Fiber-like shear-stiffening model of Eq. (S24) with $c = 10$, following the predictions up to the point where stresses diverge (when $\eta \sim 1/\sqrt{c}$). For both plots, $\nu = 0.1$ and $r_{\rm out}/r_{\rm in} = 2$.

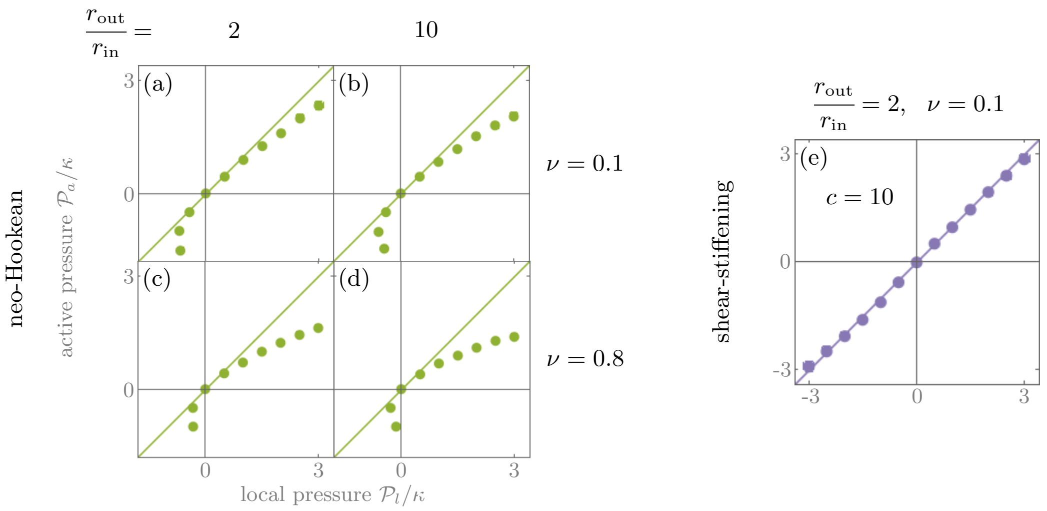

FIG. S5. Investigation of the $\mathcal{P}_a \sim \mathcal{P}_l$ relationship of Eq. (10) for $\mathcal{S}_l = 0$. The predictions at zero shear stress (*i.e.* $|\mathcal{S}_l|, |\mathcal{S}_a| < 0.01\kappa$) remain reasonably accurate up to a few $\mathcal{P}_l/\kappa$. (a-d) In the neo-Hookean case, the nonlinear terms are such that $\mathcal{P}_a < \mathcal{P}_l$ and become increasingly significant as ν and $r_{\text{out}}/r_{\text{in}}$ increase. Specifically, the agreement deteriorates from panel (a) to panels (b) and (c), to panel (d). (e) In the shear-stiffening case these nonlinear terms are first decreased up to $c \sim$ 2-5 [see Eq. (S24)] and then increased in the opposite direction (such that $\mathcal{P}_a > \mathcal{P}_l$).

small stress regime for all considered values of the Poisson's ratio ν and the ratio of the boundary radius to the active unit radius $r_{\text{out}}/r_{\text{in}}$.

We then investigate a variant of the neo-Hookean model which has the shear-stiffening behavior $G \propto \sigma_{xy}^{3/2}$ characteristic of fiber networks under large strains. Its elastic energy density reads [7, 8]

$$E = \frac{\kappa}{2}(J-1)^2 + \frac{\mu}{2c}\Big[1 - c\big(I/J - 2\big)\Big]^{-1}, \tag{S24}$$

where $J = \det(\mathbf{1} + \boldsymbol{\eta})$ and $I = \text{Tr}(\mathbf{1} + 2\boldsymbol{\varepsilon})$. Here, the shear strain threshold for the stiffening behavior corresponds to a fraction of $1/\sqrt{c}$, the strain at which the shear stress diverges. This is such that the neo-Hookean model is recovered for $c = 0$. This model still has $\kappa_1 = 1/2$, $\mu_1 = -3/2$, which corresponds to the same tendency to rectify towards contraction as in the neo-Hookean case. Indeed, c only affects higher order nonlinearities. As shown in Fig. S4(b,c) where $c = 10$, we recover Eq. (10) at small stress. But due to the shear stress divergence at finite shear strain in Eq. (S24), our predictions fail when $\mathcal{S}_l/\kappa \gtrsim 1/\sqrt{c}$.

We finally compare the dependence of $\mathcal{P}_a$ with $\mathcal{P}_l$ at zero shear stress with the weakly nonlinear prediction $\mathcal{P}_a \sim \mathcal{P}_l$. As displayed in Fig. S5, we recover the prediction for small stresses, but higher order nonlinearities induce significant deviations for $\mathcal{P}_l/\kappa$ outside of $[-0.3, 1]$. In the end, we see that the predictions of Eq. (10) relating the active and local stress components fail when either the local pressure or the local shear stress become comparable to the bulk modulus κ. Our weakly nonlinear predictions additionally fail close to stress divergences, *i.e.* when the nonlinearities become too significant compared to the linear terms.

[1] P. Wriggers, *Nonlinear Finite Element Methods*, 1st ed. (Springer, Berlin, Heidelberg, 2008).
[2] P. Ronceray and M. Lenz, Soft Matter **11**, 1597 (2015).
[3] A. E. Carlsson, Physical Review E **74**, 051912 (2006).
[4] C. S. O'Hern, L. E. Silbert, A. J. Liu, and S. R. Nagel, Phys. Rev. E **68**, 011306 (2003).
[5] M. van Hecke, Journal of Physics: Condensed Matter **22**, 033101 (2009).
[6] M. S. Alnæs, J. Blechta, J. Hake, A. Johansson, B. Kehlet, A. Logg, C. Richardson, J. Ring, M. E. Rognes, and G. N. Wells, Archive of Numerical Software **3** (2015).
[7] Y. Shokef and S. A. Safran, Physical Review Letters **108**, 178103 (2012).
[8] J. K. Knowles, International Journal of Fracture **13**, 611 (1977).
